# Author Correction: The anatomy of social dynamics in escape rooms

**DOI:** 10.1038/s41598-025-88573-5

**Published:** 2025-02-12

**Authors:** Rebeka O’Szabo, Sandeep Chowdhary, David Deritei, Federico Battiston

**Affiliations:** 1 Department of Network and Data Science, Central European University, Budapest, 1051 Hungary; 2 Laboratory for Networks, Innovation and Technology, Corvinus University, Budapest, 1093 Hungary; 3 Department of Network and Data Science, Central European University, Vienna, 1100 Austria; 4 Department of Molecular Biology, Semmelweis University, Budapest, 1085 Hungary; 5 Channing Division of Network Medicine, Harvard Medical School, Boston, 02115 USA

Correction to: *Scientific Reports* 10.1038/s41598-022-13929-0, published online 22 June 2022

The original version of this Article contained an error in the spelling of the author Rebeka O’Szabo which was incorrectly given as Rebeka O.Szabo.

The original Article has been corrected.

