# Supplementary material for: The anatomy of social dynamics in escape rooms
Source: Sci Rep. 2022 Jun 22;12:10498. doi: 10.1038/s41598-022-13929-0 (PMC9217954; doi:10.1038/s41598-022-13929-0)
Supplement: Supplementary file 1 — Supplementary Information. [file 41598_2022_13929_MOESM1_ESM.pdf]

Supplementary Information
The anatomy of social dynamics in escape rooms

Rebeka O. Szabo, Sandeep Chowdhary, David Deritei, and Federico Battiston

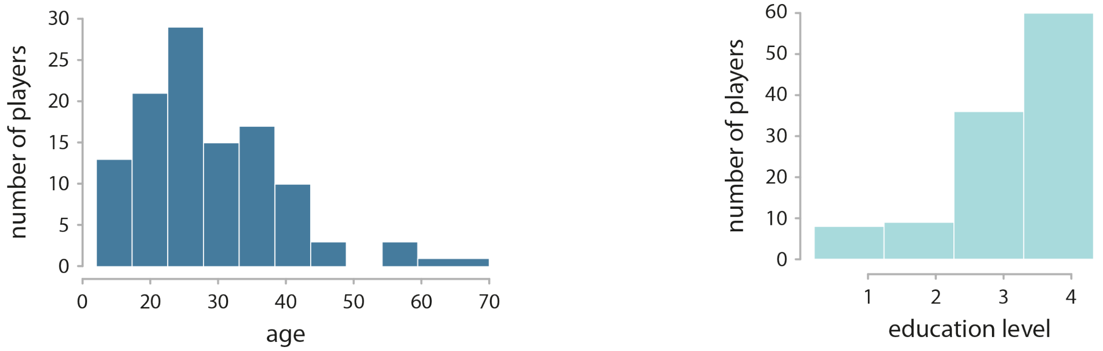

FIG. S1. **Distribution of age and education.**

As Fig. S1 shows, the majority of escape room players in our sample are young, 20-30 years old. The education level of participants is high, mainly equivalent to a diploma in higher education (value 4).
Our sample consists of 85 males and 86 female participants.

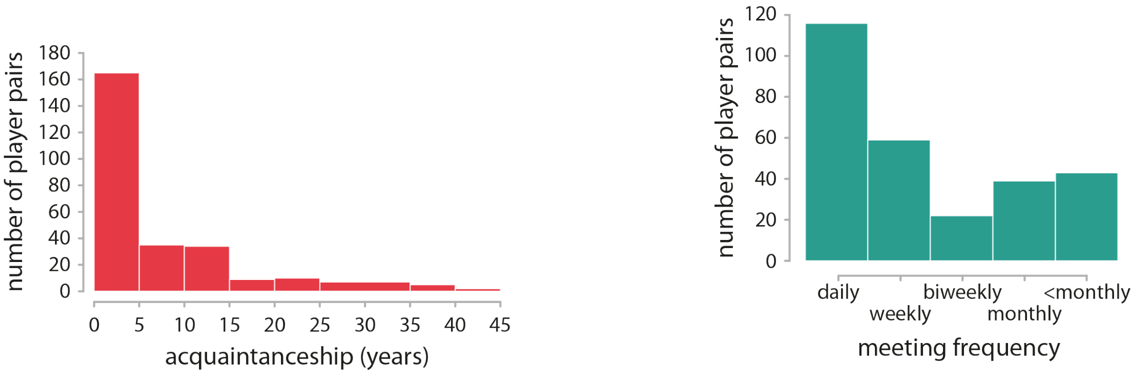

FIG. S2. **Distribution of acquaintanceship and meeting frequency.**

A significant number of participant pairs have known each other for a few years (0-5). We can see long acquaintanceships (10-15 years) in the case of a few team player pairs, while only a very few members have known each other for more than 15 years. Most of the escape room players meet on a daily basis. In general, participants meet either frequently (daily/weekly) or rarely (monthly/less than a month in our sample(Fig. S2).

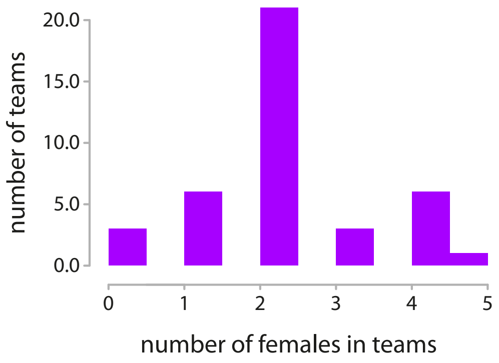

FIG. S3. **Number of females in teams.**

As Fig.R S3 shows, around half of the teams observed have two females among their members, implying gender balance (or very close to it, for the asymmetrical case of 5 individuals in a team).

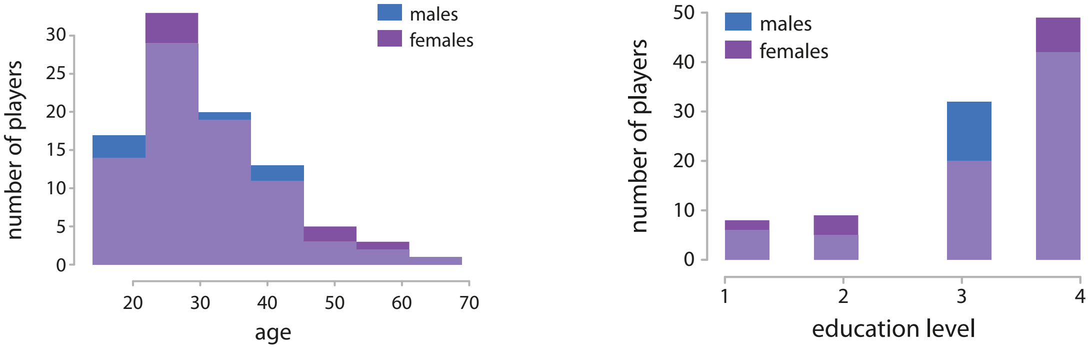

FIG. S4. **Age and education distribution by gender.**

We also observe that there are slightly more woman players in their 30's and over 40's than men. Regarding the distribution of education levels, males tend to have a slightly higher education (high school graduation), while more females can be found in the other categories such as elementary school, vocational and higher education (Fig.R S4. Nevertheless, none of these differences are statistically significant, as shown by the results of the KS test and associated p-value. As a consequence, it is extremely unlikely that these small sociodemographic differences across gender have the potential to impact our findings.

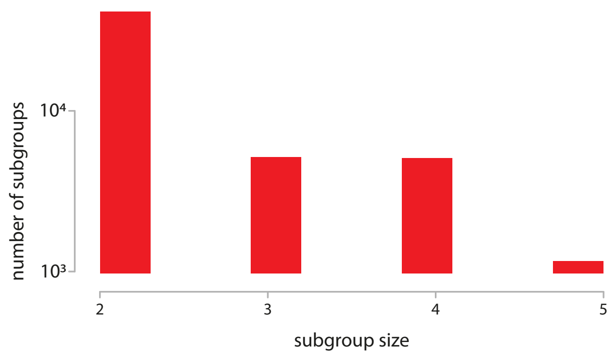

FIG. S5. **Distribution of subgroup sizes.**

In Fig.R S5 we show the size distribution of the different interactions. As displayed, most of them ($\approx 80\%$) are pairwise interactions. Yet, a significant fraction of them are group interactions ($\approx 20\%$). Interestingly, while almost no 5-person interactions are observed (not even in teams composed of 5 members), group interactions are almost equally split into interactions involving 3 and interactions involving 4 members.
